# The relationship between smartphone addiction and academic burnout among physical education teacher students: a chain mediating of meaning in life and physical exercise

**DOI:** 10.3389/fpsyg.2026.1903004

**Published:** 2026-08-10

**Authors:** Hui Ma, Lijun Liu, Tong Liu, Shuting Huang, Zhonggen Yin, Wenjie Cheng

**Affiliations:** 1 School of Marxism, Chengdu Sport University, Chengdu, Sichuan, China; 2 Sichuan Provincial Sports Museum, Chengdu, Sichuan, China; 3 School of Sports Training, Chengdu Sport University, Chengdu, Sichuan, China; 4 Chongqing Yucai Secondary School, Chongqing, China; 5 College of Physical Education and Health Management, Chongqing University of Education, Chongqing, China; 6 School of General Education, Sichuan Fine Arts Institute, Chongqing, China

**Keywords:** academic burnout, chain mediating, meaning in life, physical exercise, smartphone addiction

## Abstract

**Objective:**

Based on an integrated perspective of self-determination theory, this study examined whether smartphone addiction is associated with academic burnout among physical education teacher students through the sequential mediation of meaning in life and physical exercise.

**Methods:**

A cross-sectional design was adopted. Participants, who were physical education teacher students, were recruited using stratified sampling. Standardized scales were used to assess smartphone addiction, meaning in life, physical exercise, and academic burnout. After controlling for demographic variables such as gender and grade, a bias-corrected bootstrap method was applied to test the chain mediation effects.

**Results:**

Correlation analyses showed a significant positive association between smartphone addiction and academic burnout (total effect *β* = 0.604, *p* < 0.001). The mediation analysis indicated that smartphone addiction was indirectly associated with academic burnout through three pathways: (1) the independent mediating pathway of meaning in life (*β* = 0.058, *p* < 0.001); (2) the independent mediating pathway of physical exercise (*β* = 0.183, *p* < 0.001); and (3) the chain mediating pathway through meaning in life and then physical exercise (*β* = 0.156, *p* < 0.001). The bootstrap confidence intervals for all indirect effects did not include zero. The total indirect effect accounted for 65.56% of the total effect, indicating statistical significance for each mediating pathway.

**Conclusion:**

The study found that smartphone addiction was not only directly and positively associated with academic burnout among physical education teacher students, but also indirectly associated through the chain mediation of meaning in life and physical exercise. The results reveal a sequential psychological-behavioral chain of “technology use behavior → psychological sense of meaning → physical activity pattern → academic adjustment problem,” clarifying the sequential relationship between meaning in life and physical exercise in the association between smartphone addiction and academic burnout. Self-determination theory provides a comprehensive theoretical framework for understanding this chain mechanism, explaining different psychological stages from need satisfaction to meaning experience and from meaning experience to autonomous behavior.

## 1 Introduction

The widespread use of smartphones has brought convenience to college students’ learning and daily life, but excessive use or addiction has raised concerns (Sunday et al., 2021; Busch and McCarthy, 2021). Smartphone addiction refers to a state in which individuals have difficulty controlling their phone use, which interferes with normal life, and is negatively associated with cognitive resources, emotional regulation, and behavioral engagement (Griffiths, 2005). Physical education teacher students, as an important force in future school physical education, need to master both theoretical knowledge and motor skills, and undertake the mission of cultivating the physical and mental health of children and adolescents (Kasmalieva, 2023; Dobson, 2023). Academic burnout is not uncommon in this group, manifesting as emotional exhaustion, a sense of alienation from academic work, and reduced personal accomplishment (Maslach and Jackson, 1981), and is negatively associated with learning motivation and professional identity (Jiang and Zhu, 2024). Therefore, exploring the mechanisms underlying the association between smartphone addiction and academic burnout among physical education teacher students has important practical significance.

Previous studies have shown a positive association between smartphone addiction and academic burnout, but the internal process of this relationship remains to be further explored (Yang et al., 2024; Wang et al., 2025). Some studies have introduced single mediating variables (e.g., self-control, sleep quality) to explain the association between addiction and burnout (Kaya, 2024; Achuodho and Piko, 2025). However, the formation of academic burnout may be the result of multi-factor, multi-stage interactions, and a single mediator may obscure more complex dynamic relationships. In other words, the association may be sequentially linked to multiple mediating factors along a chain of psychological and behavioral processes. Therefore, it is necessary to construct a chain mediation model to characterize the logical sequence or hierarchy of the mediating variables.

This study attempts to build a chain mediation model and explain it using self-determination theory. The theory states that satisfaction of basic psychological needs (autonomy, competence, relatedness) is closely associated with intrinsic motivation and health behaviors (Ryan and Deci, 2000). This study speculates that meaning in life and physical exercise may be two sequential mediating factors. Meaning in life refers to the life goals and importance that an individual extracts from his or her life experiences (Steger et al., 2006), and physical exercise is an important behavior for professional learning and health regulation among physical education teacher students (Royet et al., 2025). Accordingly, this study proposes a chain mediation path: smartphone addiction → meaning in life → physical exercise → academic burnout.

It should be noted that this study adopts a cross-sectional design, aiming to explore the patterns of associations and mediating pathways among variables without making causal inferences. The research objective is to test the significance of the above chain mediation model. Theoretically, this study helps to deepen the understanding of the mechanisms linking smartphone addiction and academic burnout, and to enrich the application of self-determination theory in the field of technology addiction and academic emotions. Practically, if the model is supported, it suggests that intervention strategies could target the two sequential steps of enhancing meaning in life and promoting physical exercise, providing multi-level pathways to alleviate academic burnout among physical education teacher students, and offering preliminary evidence for future longitudinal or experimental studies.

## 2 Literature review and hypothesis development

### 2.1 Smartphone addiction and academic burnout

Smartphone addiction is widely operationalized as problematic smartphone use, characterized by loss of control, withdrawal symptoms, tolerance, and functional impairment (e.g., interference with academic or social functioning) (Kwon et al., 2013). Academic burnout results from prolonged academic stress and typically includes three interrelated dimensions: emotional exhaustion, depersonalization, and low personal accomplishment (Maslach and Jackson, 1981). In general college student samples, a positive association between smartphone addiction and academic burnout has received relatively consistent empirical support (Purnomo et al., 2020; Sadeghi Bimorgh et al., 2023; Yao et al., 2025). However, research focusing specifically on physical education teacher students is still insufficient. The academic tasks of physical education teacher students are dual in nature, involving both theoretical coursework and motor skill training with physical fitness maintenance (Choi et al., 2022); motor skill training demands higher levels of concentration, time management, and physical condition (Anguera et al., 2022). Excessive smartphone use may be associated with crowding out training time (Güler et al., 2023), disturbing sleep rhythms (Elsheikh et al., 2024), or reducing classroom attention (Liebherr et al., 2020), and in turn, be associated with various dimensions of academic burnout (Cao et al., 2018; El Barusi and Kurniawati, 2024). Nevertheless, existing studies often treat physical education students as a subsample of general college students, ignoring the possible moderating or mediating role of their professional characteristics in the relationship.

More importantly, previous studies have mainly reported the total or direct effects between smartphone addiction and academic burnout, and rarely examined whether the association is sequentially transmitted through other psychological and behavioral variables. For physical education teacher students, variables such as professional identity, meaning in life, and physical exercise habits may play sequential mediating roles. Therefore, it is necessary to first confirm whether smartphone addiction and academic burnout are positively associated in this specific group, which is the prerequisite for the subsequent chain mediation test. Based on the above analysis, this study proposes Hypothesis H1: Smartphone addiction is positively associated with academic burnout among physical education teacher students.

### 2.2 Smartphone addiction, meaning in life, and academic burnout

Meaning in life refers to an individual’s sense of significance and perception of self-importance, and is an important psychological resource for maintaining mental health and academic engagement (Steger et al., 2006). Self-determination theory suggests that satisfaction of basic psychological needs is an important source of meaning in life (Ryan and Deci, 2000). When individuals’ needs for autonomy, competence, and relatedness are satisfied, they are more likely to experience meaning in life (Lambert et al., 2013; Martela et al., 2024). Excessive smartphone use is associated with reduced real-life interpersonal interaction (Avgustis, 2023) and lower quality of life (Masaeli and Billieux, 2022). Moreover, lack of meaning in life may be associated with more negative emotional experiences and greater smartphone overuse (Qiu et al., 2022).

The relationship between meaning in life and academic burnout is also worth attention. Individuals with higher meaning in life tend to view academic work as valuable life content and are more likely to gain satisfaction from learning, thus being associated with lower levels of academic burnout (Klutsey and Mahama, 2025; Zhang et al., 2026). For physical education teacher students, professional learning involves not only theoretical knowledge but also physical practice and motor skill training, which themselves require strong intrinsic motivation. Those with higher meaning in life are more likely to perceive value and enjoyment from their physical education studies (Zhao and Yin, 2024), and are associated with lower levels of negative manifestations of academic burnout (Güngör and Sari, 2022).

However, existing studies have mostly examined the pairwise relationships between smartphone addiction and meaning in life (Wang et al., 2020; Ye et al., 2025), and between meaning in life and academic burnout separately (Güngör and Sari, 2022; Zhang and Chen, 2025), and have rarely integrated all three into one model to test the mediating role of meaning in life. Given the uniqueness of professional identity and physical practice among physical education teacher students, meaning in life may be a key explanatory variable. In other words, the association between smartphone addiction and academic burnout may be partially transmitted through the mediating pathway of meaning in life. Based on the above analysis, this study proposes Hypothesis H2: Meaning in life mediates the association between smartphone addiction and academic burnout among physical education teacher students.

### 2.3 Smartphone addiction, physical exercise, and academic burnout

Physical exercise refers to intentional, planned bodily activity (Howley, 2001). For physical education teacher students, it is both a basic component of professional competence and an important way to maintain physical and mental health (Spittle et al., 2022). From the perspective of self-determination theory, regular physical exercise is associated with satisfaction of autonomy and competence needs, because individuals need to actively plan training content and experience skill improvement in practice (Teixeira et al., 2012). Among general college students, smartphone addiction is negatively associated with the frequency and duration of physical exercise participation (Pirwani and Szabo, 2024), while physical exercise is negatively associated with academic burnout (Rosales-Ricardo and Ferreira, 2022). Specifically, higher levels of physical exercise are accompanied by lower emotional exhaustion and higher personal accomplishment (Mabele et al., 2024); this association may be related to the positive effects of physical exercise on stress regulation (Ahmed et al., 2023), self-efficacy (Wang et al., 2022), and sleep quality (Banno et al., 2018). Although these two pairwise relationships have received empirical support, evidence on whether physical exercise plays a mediating role between smartphone addiction and academic burnout is still lacking.

The special characteristics of physical education teacher students make this mediating pathway worth separate attention. Compared with students in other majors, physical education teacher students have academic tasks that include substantial physical training and skill practice, and physical exercise itself is a major part of their professional learning. Excessive smartphone use may be associated with lower regularity of physical exercise through two mechanisms: time crowding and attention distraction. For example, prolonged phone use is associated with reduced training time and inadequate post-training recovery (Gantois et al., 2021). Less physical exercise participation is associated with decreased physical fitness, diminished emotional regulation ability, and certain manifestations of academic burnout (e.g., emotional exhaustion and reduced accomplishment) (Mabele et al., 2024). Therefore, physical exercise may constitute a mediating pathway between smartphone addiction and academic burnout. Based on the above analysis, this study proposes Hypothesis H3: Physical exercise mediates the association between smartphone addiction and academic burnout among physical education teacher students.

### 2.4 Smartphone addiction, meaning in life, physical exercise, and academic burnout

The previous sections proposed the independent mediating role of meaning in life and the independent mediating role of physical exercise between smartphone addiction and academic burnout. On this basis, the present study further proposes a chain mediation mechanism, i.e., meaning in life and physical exercise may be sequentially associated with smartphone addiction and academic burnout. A large body of empirical research has shown a significant positive association between meaning in life and physical exercise in different populations. For example, a cross-sectional survey of college students showed that individuals with higher meaning in life also reported more frequent participation in physical exercise (Zhao and Yin, 2024). Similarly, positive associations between meaning in life and motivation for health behaviors as well as regular physical activity have been observed in adolescent samples (Yemiscigil and Vlaev, 2021). This pattern extends to physical education students as well; one study found that meaning in life was positively associated with persistence in physical exercise among physical education majors (Guo et al., 2023). Taken together, these findings suggest a stable positive association between meaning in life and physical exercise, providing an empirical basis for their sequential relationship.

From the perspective of self-determination theory, the above sequential association has a theoretical logical order. The degree of satisfaction of basic psychological needs (autonomy, competence, relatedness) is positively associated with meaning in life (Martela et al., 2024), and meaning in life is in turn positively associated with intrinsic motivation to engage in physical exercise (Teixeira et al., 2012). Thus, a theoretical sequential path can be proposed: smartphone addiction is associated with lower satisfaction of basic psychological needs; this need-frustration state is associated with lower meaning in life; lower meaning in life is associated with less physical exercise participation; and less physical exercise participation is associated with higher academic burnout. This chain path does not exclude other possibilities. For example, physical exercise might also be associated with higher meaning in life, or the two might have a non-sequential interactive relationship. Because this study uses a cross-sectional design, it cannot infer the temporal order of the variables; therefore, the sequential order is only a theoretical assumption.

**Figure 1:**
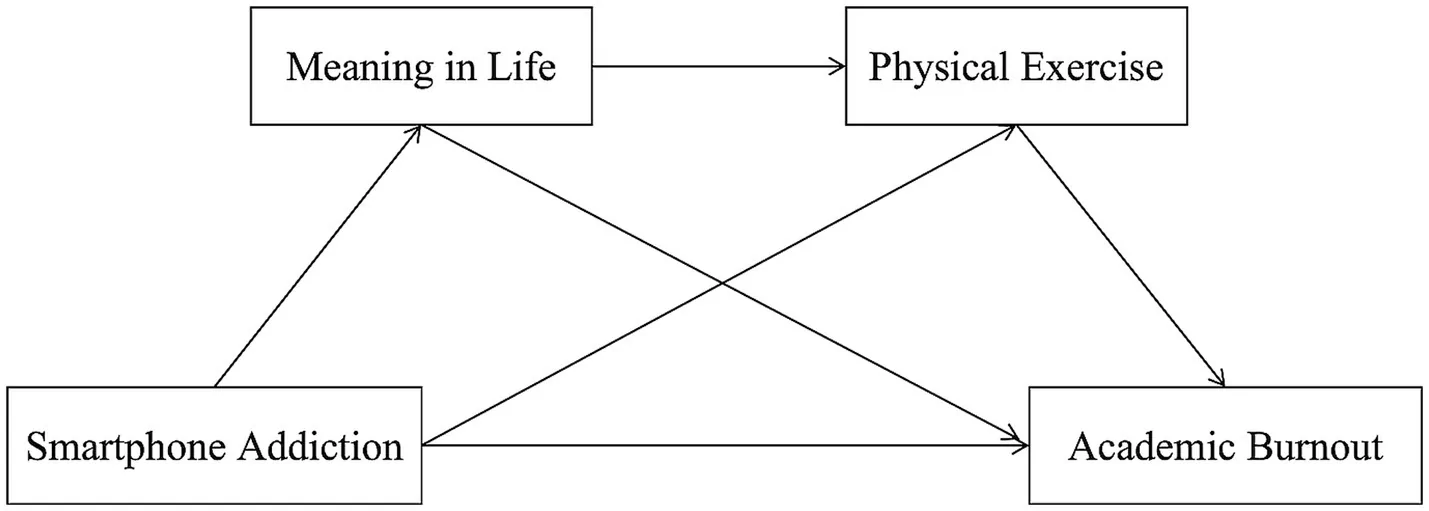
The chain mediation effect model.

Based on the above analysis, this study proposes Hypothesis H4: Meaning in life and physical exercise play a chain mediating role between smartphone addiction and academic burnout among physical education teacher students, i.e., there is a sequential association among smartphone addiction, lower meaning in life, less physical exercise, and higher academic burnout (Figure 1).

## 3 Methodology

### 3.1 Procedures and participants

This study adopted a cross-sectional design. From October 1 to October 31, 2025, a stratified sampling method was used to recruit 1,300 adult physical education teacher students from 15 provinces across five regions of China: East, Central, West, South, and North. The questionnaires were distributed online by academic advisors. Before distribution, the research team provided special training to the advisors, covering key aspects of questionnaire design such as voluntary participation procedures, withdrawal mechanisms, and confidentiality requirements.

Subsequently, the class advisors distributed the questionnaires to the physical education teacher students. Participants completed the questionnaires under the supervision of their teachers, during which the teachers explained in detail the principle of voluntary participation, the meaning of the questions, and the right to withdraw. After data collection, 108 invalid responses were excluded based on pre-established criteria: (1) abnormally short or long completion time; (2) inattentive response patterns (e.g., repetitive or contradictory answers).

The final sample included 1,192 participants, aged 18 to 24 years (mean = 19.5, SD = 1.265). Among them, 648 were male (54.36%) and 544 were female (45.64%). The grade distribution was as follows: 471 freshmen (39.51%), 342 sophomores (28.69%), 266 juniors (18.96%), and 113 seniors (9.48%). By household registration type: 580 from urban areas (48.66%) and 612 from rural areas (51.34%). The effective response rate was 91.69%. Exclusion criteria included: (1) severe physical illness; (2) history of mental illness; (3) receipt of psychological therapy within the past 3 months.

Data were collected through an online platform^1^ using an electronic questionnaire. The questionnaire consisted of five sections: demographic information (grade, gender, height, weight), the Smartphone Addiction Scale (SAS-SV), the Meaning in Life Questionnaire (MLQ), the Physical Exercise Scale (PA), and the Academic Burnout Scale (ALBI). Before data collection, all participants signed an electronic informed consent form. After data collection, all information was anonymized to ensure anonymity. The anonymized data set will be properly stored in accordance with institutional ethical guidelines and data management policies, and will be available for scientific research use for at least 5 years after publication. Qualified researchers may request access to the data but must sign a formal data-sharing agreement to ensure the privacy of participants and comply with the original informed consent terms.

### 3.2 Ethics approval and consent to participate

This study strictly followed the ethical principles of the Declaration of Helsinki. The research protocol was approved by the Ethics Committee of Chengdu Sport University (approval number: CTYLL2025235). All participants signed an electronic informed consent form through the online platform before formal participation. The informed consent form detailed the purpose of the study, the principle of voluntary participation, confidentiality agreements, and the right to withdraw from the study at any time without any liability. To ensure the privacy of participants, all data were anonymized during collection and analysis, and no personal identifying information was retained in the final dataset.

### 3.3 Measurement tools

#### 3.3.1 Smartphone addiction scale

Smartphone addiction was measured using the Smartphone Addiction Scale developed by Kwon et al. (2013). The scale is unidimensional and consists of 10 items (e.g., “I have delayed planned study or work because of smartphone use,” “I have had difficulty concentrating on study or work because of smartphone use”). Each item is rated on a 6-point scale (1 = “strongly disagree,” 6 = “strongly agree”), with higher scores indicating less agreement with the item description. Confirmatory factor analysis was conducted to evaluate the construct validity of the scale. Model fit indices indicated good fit: χ^2^/df = 1.241, GFI = 0.993, AGFI = 0.989, RMSEA = 0.014 (90% CI [0.000, 0.027]). In this study, the Cronbach’s *α* coefficient for this scale was 0.923.

#### 3.3.2 Meaning in life questionnaire

Meaning in life was measured using the Meaning in Life Questionnaire (MLQ) developed by Steger et al. (2006). The scale includes two dimensions with a total of 10 items: presence of meaning (5 items, e.g., “I understand the meaning of my life”) and search for meaning (5 items, e.g., “I am looking for something that makes my life meaningful”). Each item is rated on a 7-point scale (1 = “completely disagree,” 7 = “completely agree”), with higher scores indicating greater agreement with the item description. Confirmatory factor analysis was conducted to evaluate the construct validity of the scale. Model fit indices indicated excellent fit: χ^2^/df = 0.990, GFI = 0.994, AGFI = 0.991, RMSEA = 0.000 (90% CI [0.000, 0.021]). In this study, the Cronbach’s *α* coefficient for this scale was 0.826.

#### 3.3.3 Physical exercise scale

This study used the Physical Exercise Behavior Scale revised by Deqing (1994) to measure participants’ exercise status over the past month. The scale includes three factors: exercise intensity, exercise duration, and exercise frequency. Exercise intensity and frequency are rated on a 5-point scale, and exercise duration is rated on a 4-point scale. The amount of exercise was calculated using the formula: “exercise amount = intensity × duration × frequency.” Higher scores indicate greater exercise amount per session. In this study, the Cronbach’s α coefficient for this scale was 0.951.

#### 3.3.4 Academic burnout scale

Academic burnout was measured using the Adolescent Learning Burnout Scale developed by Yan and Xiao-Yang (2010), based on Maslach’s Burnout Inventory (Maslach and Leiter, 2000). The scale includes three dimensions with a total of 16 items: psychosomatic exhaustion (4 items, e.g., “I feel empty lately and do not know what to do”), academic alienation (5 items, e.g., “I feel that I do not understand anyway, so it does not matter whether I study or not”), and low sense of accomplishment (7 items, e.g., “I am able to cope with exams well”). Each item is rated on a 5-point scale (1 = “very consistent,” 5 = “very inconsistent”), with higher scores indicating less consistency with the item description. Confirmatory factor analysis was conducted to evaluate the construct validity of the scale. Model fit indices indicated good fit: χ^2^/df = 0.881, GFI = 0.991, AGFI = 0.988, RMSEA = 0.000 (90% CI [0.000, 0.010]). In this study, the Cronbach’s α coefficient for this scale was 0.834.

### 3.4 Data analysis

The data analysis procedure consisted of three steps.

First, common method bias was assessed using the Harman single-factor test.

Second, descriptive statistics and correlation analysis were performed. Means, standard deviations, and Pearson correlation coefficients were calculated for all variables, including smartphone addiction, meaning in life, physical exercise, and academic burnout.

Third, chain mediation analysis was conducted using the PROCESS macro (Version 4.0) in SPSS 25.0. A chain mediation model was constructed to test the pathway from smartphone addiction to academic burnout through the mediating effects of meaning in life and physical exercise. Gender, grade, and monthly living expenses were included as covariates. The bias-corrected bootstrap method with 5,000 iterations and 95% confidence intervals was used to test the indirect effects.

## 4 Results

### 4.1 Common method bias test

Because the questionnaire data were obtained through self-reports from participants, the Harman’s single-factor test was used to examine potential common method bias. An exploratory factor analysis was conducted on all items of the Smartphone Addiction Scale, Meaning in Life Questionnaire, Physical Exercise Scale, and Academic Burnout Scale. Principal component analysis was used to extract common factors, and the first unrotated factor was examined. The results showed that the first factor explained 36.675% of the variance, which is below the critical threshold of 40%, indicating that no significant common method bias was present in this study.

### 4.2 Descriptive statistics and correlations

Descriptive statistics and Pearson correlation analyses were performed for the four variables (smartphone addiction, meaning in life, physical exercise, academic burnout) and are presented in Table 1. All variables showed significant intercorrelations.

**Table 1:** Descriptive statistics and correlation analysis of variables.

Variable	M	SD	Smartphone addiction	Meaning in life	Physical exercise	Academic burnout
Smartphone addiction	38.380	10.955	1			
Meaning in life	46.000	9.013	−0.599**	1		
Physical exercise	74.930	29.627	−0.615**	0.671**	1	
Academic burnout	49.940	9.600	0.689**	−0.674**	−0.848**	1

**p < 0.01.

Smartphone addiction was positively correlated with academic burnout (r = 0.689, *p* < 0.01). Smartphone addiction was negatively correlated with meaning in life (r = −0.599, *p* < 0.01) and with physical exercise (r = −0.615, *p* < 0.01). Meaning in life was positively correlated with physical exercise (r = 0.671, *p* < 0.01) and negatively correlated with academic burnout (r = −0.674, *p* < 0.01). Physical exercise was negatively correlated with academic burnout (r = −0.848, *p* < 0.01).

### 4.3 Chain-mediated effects analysis

To examine the predictive associations of smartphone addiction, meaning in life, and physical exercise with academic burnout, hierarchical regression analyses were conducted. Smartphone addiction, meaning in life, and physical exercise were entered as independent variables and academic burnout as the dependent variable (Table 2).

**Table 2:** Regression analysis of smartphone addiction, meaning in life, and physical exercise on academic burnout.

Variant	Academic burnout			
	β	T	F	R^2^
Smartphone addiction	0.689	32.794***	1075.473	0.474
Meaning in life	−0.674	−31.448***	988.988	0.453
Physical exercise	−0.848	−55.087***	3034.591	0.718

***p < 0.001.

The results showed that smartphone addiction was positively associated with academic burnout (*β* = 0.689, t = 32.473, *p* < 0.001); meaning in life was negatively associated with academic burnout (*β* = −0.674, t = −31.448, *p* < 0.001); and physical exercise was negatively associated with academic burnout (*β* = −0.848, t = −55.087, *p* < 0.001).

This study took smartphone addiction (X) as the independent variable, academic burnout (Y) as the dependent variable, and meaning in life (W1) and physical exercise (W2) as mediating variables. After controlling for demographic variables (gender, grade, monthly living expenses), the chain mediating effect of meaning in life and physical exercise on the relationship between smartphone addiction and academic burnout was tested. The PROCESS macro (Version 4.0) in SPSS 25.0 was used for the analysis. Model 6 of the PROCESS macro was employed for hypothesis testing, and the bias-corrected bootstrap method with 5,000 resamples and 95% confidence intervals (CIs) was used to test the chain mediation effects.

As shown in Table 3, smartphone addiction was positively associated with academic burnout among physical education teacher students (β = 0.674, *p* < 0.001; 95% CI [0.568, 0.640]), indicating that higher smartphone addiction scores were accompanied by greater severity of academic burnout. This supported Hypothesis 1. The association between smartphone addiction and academic burnout was transmitted through the mediating pathway of meaning in life (*p* < 0.001; 95% CI [0.036, 0.081]); that is, higher smartphone addiction was associated with lower meaning in life, and lower meaning in life was associated with higher academic burnout, thus supporting Hypothesis 2. Physical exercise mediated the association between smartphone addiction and academic burnout (*p* < 0.001; 95% CI [0.155, 0.211]); that is, higher smartphone addiction was associated with less physical exercise, and less physical exercise was associated with higher academic burnout, supporting Hypothesis 3. Finally, meaning in life and physical exercise played a chain mediating role between the two (*p* < 0.001; 95% CI [0.135, 0.177]); that is, higher smartphone addiction was associated with lower meaning in life, lower meaning in life was associated with less physical exercise, and less physical exercise was associated with higher academic burnout, thus supporting Hypothesis 4.

**Table 3:** Mediation effect values and effect sizes.

Path	Effect	BOOT SE	BOOT LLCI	BOOT ULCI	Relative mediation effect
Total effect	0.604***	0.018	0.568	0.640	100%
Direct effect	0.207***	0.016	0.175	0.239	34.272%
Total indirect effect	0.396***	0.015	0.367	0.427	65.563%
Indirect effect 1 (meaning in life)	0.058***	0.011	0.036	0.081	9.603%
Indirect effect 2 (physical exercise)	0.183***	0.014	0.155	0.211	30.298%
Indirect effect 3 (meaning in life and physical exercise)	0.156***	0.011	0.135	0.177	25.828%

Indirect effect 1: Smartphone addiction → Meaning in life → Academic burnout.

Indirect effect 2: Smartphone addiction → Physical exercise → Academic burnout.

Indirect effect 3: Smartphone addiction → Meaning in life → Physical exercise → Academic burnout.

***p < 0.001.

**Figure 2:**
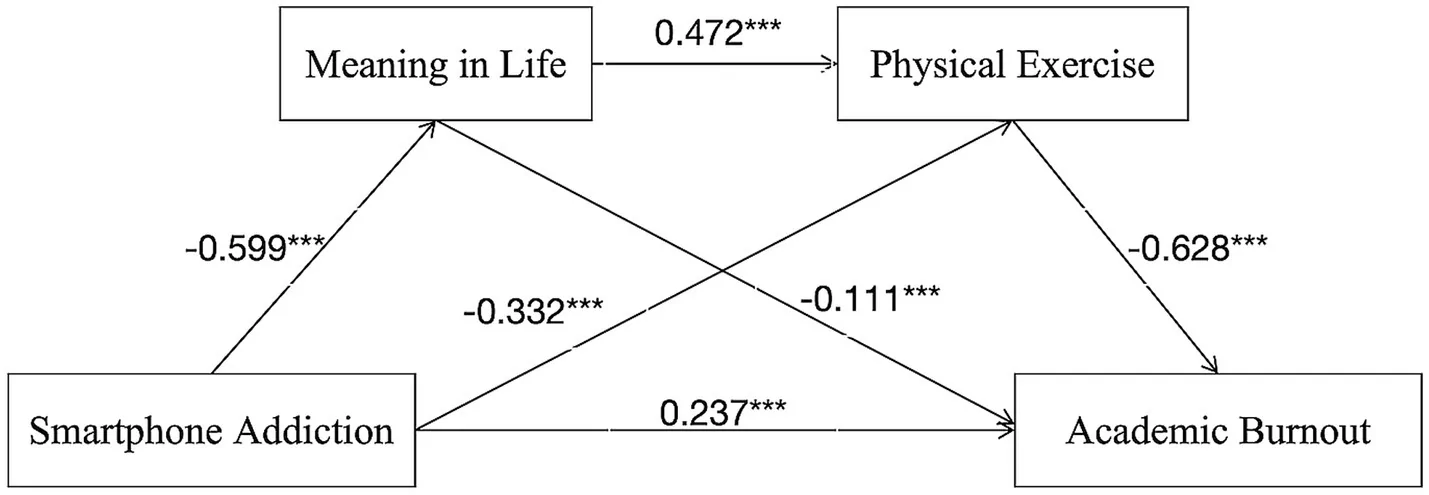
Model of chain mediating roles of meaning in life and physical exercise between smartphone addiction and academic burnout.

In addition, the effect sizes of the three mediating pathways and the total effect were all statistically significant (*p* < 0.001). The total effect was 0.604, of which the direct effect was 0.207 (34.272% of the total effect) and the indirect effect was 0.396 (65.563% of the total effect). Among the mediating pathways, Pathway 1 (smartphone addiction → meaning in life → academic burnout) had an effect size of 0.058 (9.603% of the total effect); Pathway 2 (smartphone addiction → physical exercise → academic burnout) had an effect size of 0.183 (30.298% of the total effect); and Pathway 3 (smartphone addiction → meaning in life → physical exercise → academic burnout) had an effect size of 0.156 (25.828% of the total effect). The pathways are illustrated in Figure 2. Moreover, the multicollinearity test (Table 4) showed that all variance inflation factor (VIF) values were below 5, indicating no multicollinearity issue. The Durbin-Watson (D-W) value was approximately 2, suggesting no autocorrelation in the model.

**Table 4:** Linear regression analysis results.

Variable	Unstandardized coefficients		Standardized coefficients	t	*p*	Collinearity statistics	
	B	Std. error	Beta			VIF	Tolerance
Constant	62.642	1.321	-	47.409	0.000	-	-
Smartphone addiction	0.207	0.016	0.237	12.703	0.000	1.791	0.558
Physical exercise	−0.203	0.007	−0.628	−31.214	0.000	2.086	0.479
Meaning in life	−0.118	0.021	−0.111	−5.585	0.000	2.025	0.494
R2	0.770						
*R2*adj	0.769						
F	1323.240***						
D-W	1.987						

***p < 0.001.

## 5 Discussion

This study aimed to explore the mechanisms underlying the association between smartphone addiction and academic burnout among physical education teacher students, with a focus on the chain mediating role of meaning in life and physical exercise. The results supported the initial theoretical hypotheses. It was found that smartphone addiction was not only directly and positively associated with academic burnout, but also linked through three indirect pathways: the independent mediating pathway of meaning in life, the independent mediating pathway of physical exercise, and the sequential mediating pathway of meaning in life and physical exercise. This finding indicates multiple potential channels of association between smartphone addiction and academic burnout.

### 5.1 Main findings

This study found a significant positive association between smartphone addiction and academic burnout among physical education teacher students (total effect *β* = 0.604), which is consistent with previous findings. Previous studies have reported that excessive smartphone use is positively associated with inadequate academic engagement and emotional exhaustion among college students (Hidalgo-Fuentes, 2022; Paterna et al., 2024). The present study again supported the stability of this association in a special group with dual academic tasks (theoretical courses and motor skill training), extending previous findings from general college students to this specific population, and providing empirical support from physical education teacher students for smartphone addiction as a correlate of academic burnout.

Regarding the mediating role of meaning in life, this study found that meaning in life partially mediated the association between smartphone addiction and academic burnout (indirect effect *β* = 0.058, accounting for 9.60% of the total effect). This finding is consistent with self-determination theory, which states that satisfaction of basic psychological needs is positively associated with meaning in life (Martela et al., 2024; Marwa and Xiaosong, 2023). Previous studies have examined the pairwise relationships between technology addiction and meaning in life (Wang et al., 2023; Bulut, 2026), as well as between meaning in life and academic adjustment (Aruta et al., 2022; Kritikou and Giovazolias, 2022). Building on this prior work, the present study further introduced meaning in life into the framework linking smartphone addiction and academic burnout, revealing that meaning in life is not only directly associated with academic burnout but also serves as an important explanatory pathway for the association between smartphone addiction and academic burnout, thereby extending the application of self-determination theory in the field of technology addiction and academic emotions.

Regarding the mediating role of physical exercise, this study found that physical exercise also partially mediated the association between smartphone addiction and academic burnout (indirect effect *β* = 0.183, accounting for 30.30% of the total effect), making it the largest contributor among the three indirect pathways. Surveys of college students have found that smartphone use time is negatively associated with physical exercise frequency (Ning et al., 2025), and that physical exercise is positively associated with mental health (White et al., 2024). A recent review also indicated that interventions based on satisfaction of basic psychological needs are closely associated with adolescents’ active participation in sports (Barbosa Cano and Gomez-Baya, 2025). Building on these studies, the present study included physical exercise as a mediator in the relationship model between smartphone addiction and academic burnout, revealing that smartphone addiction may be associated with academic burnout through the behavioral pathway of physical exercise. This provides a new behavioral-level explanatory pathway for understanding how smartphone addiction is associated with academic burnout, and compensates for the limitation of previous studies that focused mainly on cognitive and emotional factors while paying insufficient attention to behavioral mediators.

This study also found that meaning in life and physical exercise formed a complete sequential transmission pathway between smartphone addiction and academic burnout (indirect effect *β* = 0.156, accounting for 25.83% of the total effect). This finding means that smartphone addiction is associated with academic burnout not only through the separate channels of meaning in life or physical exercise, but also through the chain path of “smartphone addiction → lower meaning in life → less physical exercise → higher academic burnout.” The identification of this pathway suggests a potential sequential connection between the two mediating variables.

Theoretically, the ordering of meaning in life before physical exercise follows the internal logic of self-determination theory: need satisfaction fosters a global sense of meaning, which in turn energizes autonomous behaviors (Ryan and Deci, 2000). Meaning in life represents a cognitive-evaluative orientation toward one’s existence, whereas physical exercise is a concrete behavioral manifestation of that orientation. Thus, a diminished sense of meaning plausibly precedes reduced exercise participation, as purpose appraisals shape motivational states that guide subsequent actions (Teixeira et al., 2012). This ordering provides a coherent theoretical account for the observed chain path.

Previous studies have often examined meaning in life and physical exercise as parallel or independent mediators. For example, some studies have found that both meaning in life and health behaviors are associated with psychological adjustment (Labrague, 2021; Czyżowska and Gurba, 2022; Altier et al., 2025). The present study further suggests the possible sequential relationship between the two, suggesting that psychological sense of meaning and behavioral patterns may have a sequential association, together forming a psychological-behavioral chain from technology addiction to academic burnout, and providing a more detailed perspective for understanding the complex pathways linking smartphone addiction and academic burnout.

### 5.2 Theoretical contributions and implications

The core finding of this study is that meaning in life and physical exercise may constitute a sequential mediating pathway between smartphone addiction and academic burnout. The theoretical contribution of this result lies in identifying a potential progressive pathway of “psychological sense of meaning → behavioral pattern,” suggesting a potential logical order of the two mediating variables and their integrated explanatory framework.

First, the order of the chain path itself has important psychological significance. This study found that smartphone addiction was associated with lower meaning in life, lower meaning in life was associated with less physical exercise, and less physical exercise was associated with higher academic burnout. This order suggests that an individual’s subjective perception of life purpose may have a theoretical logical precedence relative to behavioral engagement. Although previous studies have shown a significant association between meaning in life and physical exercise, most have used cross-sectional designs, making it difficult to determine the causal direction (Zhao and Yin, 2024; Guo et al., 2024). Building on this, the present study, through a combination of theory-driven and empirical testing, proposed that meaning cognition may have a theoretical logical precedence over behavioral engagement. This order is consistent with the basic logic of self-determination theory, which holds that satisfaction of basic psychological needs is associated with meaning experience (Martela et al., 2024), and meaning experience is in turn associated with autonomous behavior (Guo et al., 2024). Therefore, the sequence identified in this study may provide a more logical explanatory framework for understanding the internal relationship between meaning in life and physical exercise.

Second, the chain mediation model achieves an organic integration of the internal logic of self-determination theory. Self-determination theory provides a theoretical tool for understanding the associations among basic psychological needs satisfaction, meaning in life, and autonomous behavior. The theory emphasizes that satisfaction of autonomy, competence, and relatedness needs is closely associated with intrinsic motivation and health behaviors (Ryan and Deci, 2000). Previous studies have often separately examined need satisfaction and meaning (Martela et al., 2024), or meaning and behavior (Zhao and Yin, 2024), and rarely attempted to incorporate all three into a single sequential model. This study used meaning in life as a connecting node between need satisfaction and physical exercise, linking the first and second halves of the chain path, and together forming a potential explanatory framework from technology addiction to meaning cognition, to behavioral engagement, and finally to academic burnout. The effect size distribution showed that the pathway related to behavioral autonomy (physical exercise alone, about 30%) contributed the most, while the pathway related to meaning alone (about 10%) contributed less. This difference suggests that when self-determination theory is applied to explain academic burnout, the behavioral mechanism may differ from the purely cognitive mechanism. This theoretical integration attempt responds to recent calls for deepening the application of self-determination theory (Howard et al., 2025; Spitzer et al., 2025), and provides a more inclusive analytical perspective for understanding complex psychological-behavioral problems.

Third, the identification of the chain mediation model may help to interpret inconsistencies or ambiguities in previous studies. Previous studies on the relationship between smartphone addiction and academic burnout have either found varying effect sizes for the direct association (Jin et al., 2026; Iftikhar et al., 2022) or unstable effects of mediating variables (Song et al., 2025; Chen et al., 2023). The chain model of the present study suggests that such inconsistencies may arise from simplified handling of intermediate processes. If only a single mediator, such as meaning in life or physical exercise alone, is examined, the sequential association between the two variables may be overlooked, and the cumulative effect of smartphone addiction through the complete psychological-behavioral chain (chain path contribution about 26%, while meaning in life alone contributed only about 10%) may be underestimated. In addition, the findings of this study are consistent with several research viewpoints suggesting that the relationship between technology addiction and academic adjustment is associated with multiple psychological and behavioral factors (Tülübaş et al., 2023; Han et al., 2025). The present study further suggests a potential sequential combination of meaning in life and physical exercise, providing a more specific path description for understanding the pathways through which smartphone addiction may be associated with academic burnout. At the same time, the results are consistent with observations that some interventions have limited effects, suggesting that intervention approaches may need to shift from a single focus to a chain-based, multi-node comprehensive consideration.

### 5.3 Practical significance and application prospects

The chain association path identified in this study provides exploratory reference directions for the prevention of academic burnout and the promotion of mental health among physical education teacher students. It is important to note that the following practical implications are based on cross-sectional association data and are exploratory hints; the specific effects of interventions need to be tested by future experimental studies.

For smartphone addiction, educators might consider supporting students in establishing balanced digital use habits without mandating complete abstinence. For meaning in life, values clarification exercises, volunteer service, and teaching practice may be associated with students’ intrinsic identification with the profession and their subjective perception of life purpose. The transition from meaning to exercise deserves particular attention: when low levels co-occur, integrating intrinsic motivation concepts into physical education with an emphasis on health and enjoyment over task completion might be associated with a lower risk of behavioral withdrawal. Finally, for insufficient exercise and academic burnout, diversifying optional physical activities, combining academic tutoring with stress management, and establishing early referral channels might be associated with greater coping flexibility and might be associated with reduced over-reliance on maladaptive behaviors such as excessive phone use.

For the end of the chain, insufficient physical exercise and academic burnout, practical efforts could focus on cultivating physical exercise habits and expanding academic support strategies. By designing diverse and optional physical activities, the intrinsic appeal of participating in exercise could be increased. At the same time, combining academic tutoring and stress management courses could help students enrich their strategic choices for coping with academic stress, reducing dependence on a single coping method (such as excessive phone use). For students who show signs of academic burnout, early identification and professional referral channels could be attempted.

In summary, the effect size ranking from this study provides a reference direction for prioritizing practical interventions. Steps related to physical exercise (both alone and in the chain path) contributed more and could be given priority attention. The psychological-to-behavioral transition step in the chain path was secondary. Interventions that solely enhance meaning in life should be carefully evaluated for their standalone effect, and it is suggested to combine them with behavioral activation. Because this study is cross-sectional, all the above suggestions need to be tested by longitudinal or experimental studies before being translated into specific intervention protocols.

### 5.4 Research limitations and future directions

This study has several limitations. First, the cross-sectional design cannot infer the temporal order of the variables. The sequential path of “meaning in life → physical exercise” assumed by the chain model is mainly based on theoretical derivation, and its directionality needs to be further tested by longitudinal or experimental studies. Second, the direct effect still accounted for about 34% of the total effect, suggesting that there may be other unmeasured mediating variables (such as self-control, sleep quality, social support) or alternative models (such as physical exercise as a preceding variable and meaning in life as a subsequent variable). Future studies could conduct model comparisons to determine better fit. Third, moderating variables were not examined. The effect sizes for each path are average estimates based on the full sample and may mask differences across subgroups such as gender, grade, and professional identity level. Future studies could introduce moderated mediation models to clarify the boundary conditions of the chain path. Fourth, the sample consisted of physical education teacher students, whose physical exercise is professionally mandatory, which may be related to the effect size of the physical exercise path in this study. Generalization of the conclusions to general college students or other occupational groups should be made with caution. Fifth, self-report questionnaires were used. Although statistical tests showed that common method bias was within an acceptable range, future studies could incorporate behavioral data (such as mobile phone screen time and activity tracker records) to improve objectivity of measurement. In addition, the practical value of the chain path needs to be tested by intervention studies. Future studies could design randomized controlled trials to compare the intervention effects of different strategies.

## 6 Conclusion

From an integrated perspective of self-determination theory, this study examined the mechanisms underlying the association between smartphone addiction and academic burnout among physical education teacher students, with a focus on the chain mediating role of meaning in life and physical exercise. The results showed that smartphone addiction was both directly and indirectly associated with academic burnout through the sequential mediation of meaning in life and physical exercise. This chain path suggests a psychological-behavioral sequence from technology use to meaning cognition, to physical activity, and finally to academic adjustment, offering an integrated perspective rooted in self-determination theory.

At the practical level, the findings provide exploratory reference directions for the prevention of academic burnout and promotion of mental health among physical education teacher students. Mental health education could focus on identifying and guiding smartphone use behaviors, helping students establish balanced digital use habits. It could also try to enhance students’ meaning in life and strengthen their intrinsic identification with the physical education profession. It could pay attention to maintaining and promoting physical exercise, guiding students to establish exercise motivation oriented toward health and enjoyment. At the same time, it is necessary to maintain necessary attention to early signs of academic burnout. It should be noted that this study used a cross-sectional design, and all findings are covariations and association paths among variables; temporal order and causal relationships cannot be inferred. Future studies could adopt longitudinal designs to further test the temporal relationships among the variables, and explore the boundary conditions of the chain association in different majors and cultural backgrounds, in order to further reveal the dynamic patterns of the association between smartphone addiction and academic burnout.

## Data Availability

The original contributions presented in the study are included in the article/Supplementary material, further inquiries can be directed to the corresponding author.

## Footnotes

1
https://www.wenjuan.com/s/UZBZJvYWmq/
